# Supplementary material for: Study Protocol: Strategies and Techniques for the Rehabilitation of Cognitive and Motor Deficits in Patients with Multiple Sclerosis
Source: NeuroSci. 2022 Jul 12;3(3):395–407. doi: 10.3390/neurosci3030029 (PMC11523688; doi:10.3390/neurosci3030029)
Supplement: Supplementary file 1 [file neurosci-03-00029-s001.zip › neurosci-1787520-supplementary.pdf]

Supplementary material

Motor Rehabilitation Training

Diversified and specific rehabilitation sessions will be planned for each patient, paying particular attention to motor disabilities of the lower limbs, most commonly observed and complained of by almost all patients with MS. In this regard, exercises will mainly be applied to promote: increased range of motion, muscle strength, postural control and sensitivity, and decreased spasticity, balance disorders, coordination problems and motor difficulties which are often presents in walking and in the transitions sit to stand and vice versa.

Some of the proposed exercises will be cited and described below:

<u>Exercise to increase range of motion</u>
Passive mobilization; active-assisted mobilization; selective activation of multiple joints, such as:

- HIP (coxo-femoral joint): in flexion, positioning the patient in supine or lateral decubitus; in extension with patient in lateral or prone position; internal and external rotation both supine and prone; adduction and abduction; circling movements.

- KNEE: in flexion-extension in supine position; selective movement of the patellofemoral joint through a vertical and transverse translation.

- ANKLE (tibio-tarsal joint) in flexion-extension.

- FOOT: in inversion and eversion; mobilization of the talus-calcaneal joint following the oblique direction of Henke's axis; mobilization of the tarsometatarsal and metatarsal-phalangeal joints in flexion-extension in supine decubitus, keeping the heel as a fixed point resting on the bed.

- PELVIS: in supine decubitus, mobilization in opening and closing, anteversion and retroversion; in lateral decubitus, anterior and posterior elevation movements.

- SPINE: mobilization of the dorsal and lumbar spine both in supine and in lateral decubitus.

Mobilization will also be used to inhibit spasticity of the lower limbs, to reduce painful symptoms in hypomobile segments , to allow for a better gait pattern and, consequently, to reduce the risk of falls.

<u>Exercises for the inhibition of spasticity of the lower limbs and selective and global lengthening</u>

- Load exercises: the "bridge" (to inhibit the extensor synergy of the lower limbs), lifting the pelvis by flexing the knees with the feet resting on the bed, trying not to compensate by using the trunk extensors. To make the load more perceived, two variants will becarried out: 1) crossing a lower

limb over the affected one, checking that the knee of the supporting limb did not go inside and 2) lifting (flexion of the hip and the knee) the contralateral limb to the supporting one;

- Selective stretching through an isotonic concentric contraction, to obtain a faster relaxation. In case of pain, but also to obtain a more lasting release, the isometric contraction will be also used in combination. Following each short isotonicconcentric contraction, the subject will be asked for an isometric contraction of the antagonist kinetic chain (from six to fifteen seconds) and then a relaxation followed by a deep inhalation. In the expiratory phase, the muscle or kinetic chain will be stretched as much as possible and slowly, maintaining the stretching for at least ten to twenty seconds. Finally, the elongation obtained will be fixed with an isometry of the agonist chain. Mainly, the pelvitrocanteric muscles, adductor muscles, hip extensors and knee flexors, and plantar flexors will be stretched.

<u>Exercises for static balance deficits</u>

The ability to maintain balance is directly proportional to the width of the support base and inversely proportional to the height of the center of gravity. Exercises in pyramidal progression will be proposed, in order to identify the level that put the patient in difficulty and then go to solve it, improving the balance deficit.

For each position, starting from the simplest and progressing towards the more complex, we will proceed to:

- Take advantage of a strengthening technique, exerting pushes with gradual resistance mainly at the level of the upper and lower limbs, with the aim of developing in the patient the ability to maintain the contraction over time and therefore teaching to isometrically contract the muscles and to fix the movement to have more stability. Being a technique that works in apnea, it is necessary to give adequate recruitment times so as not to cause excessive effort.

- Perform exercises aimed at training proprioception by closing the eyes and using unstable aids, such as oscillating platforms, balls, trolley.

The exercises in progression will be proposed in the various decubitus, in particular:

- In supine decubitus, we will start from the "star" position. Once all the positions have been stabilized following the prerequisite logic of the pyramidal progression, the patient will be reach the position of the bridge in monopodalic mode, with a trolley under the limb loading and the arms flexed at 90 ° with hands crossed. The same exercise will be proposed by raising the center of gravity to the maximum, making the patient maintain the position of the "table", that is the position similar to the bridge, but maintaining support only with the hands and feet.

- In lateral decubitus, it will be required to reach and maintain the position starting from the supine decubitus, and subsequently it will be

required to abduct first the upper limb, then the lower limb and then both limbs at the same time. The same progression will be performed first resting on the elbow, and then on the hand.

- By placing the patient on the mat, it will be required to maintain the quadrupedal position in support with all 4 limbs, then flexing respectively first an upper limb and then the contralateral. Subsequently, the hip extension of a lower limb along the sagittal plane (first with one limb and then with the other one) and finally, the flexion of the right upper limb simultaneously with the extension of the left lower limb and vice versa. To increase the difficulty, the unstable aid will be placed under the hand.

- Balance will be sought without help or support even from the kneeling position. With the upper limbs (shoulder joint) flexed at 90° with the elbows extended, arranged along the sagittal plane and with the hands crossed between them, exercises will be carried out to reach external targets in the different planes of space. Still in the kneeling position, the patient will be urged to throw balls into circles, used as if they were baskets, first with one upper limb and then with the other. The same exercises will be then proposed both in the position of "cavalier servente" and in the standing position, complicating the exercise by means of aids that generated instability.

- Once the upright position will be reached, the patient will be instructed to slowly decrease the support base (feet together, semi-tandem position of the feet, tandem position, monopodalic position). The same progression will be requested on an oscillating platform and with the eyes closed.

- In an upright position, specific tasks will be required, such as grabbing a ball thrown by the therapist in different directions, while keeping the feet within the same support base. This exercise will also be performed, in succession, on an oscillating platform.

<u>Global and analytical muscle strengthening exercises of the hypovalid muscles</u>

In particular, the training will be focused on improvement of the "core stability", understand as the musculature that surrounds the abdomen and pelvis, including the abdominals, gluteus and paravertebral muscles, the diaphragm as roof and the pelvic floor and the hip muscles as a basis. In this case, to increase the difficulty of the exercise, the therapist will stabilize the position by inducing isometric contractions, will reduce the support base and will raise the center of gravity.

Here are some of the exercises:

- Middle gluteus strengthening exercises in the following ways: 1) with the patient in lateral decubitus, requiring abduction and internal rotation movements of the hip 2) with the patient kneeling on a carpet, requesting a shift, first forward and then back;

- In the supine position, placing the lower limbs on a Bobath ball (hips flexed and knees extended) the patient will be asked to make oscillations with the pelvis and with the lower limbs to the right and to the left, keeping the trunk still, without support of the upper limbs and without the lower limbs slipping from the balloon. The same exercise will be required by placing the ball closer to the body, with an approximately 90° flexion of the hips and knees;

- Sitting on the edge of the bed and with the upper limbs crossed in front of the chest, the patient will have to extend the trunk (as if he will be to lie down on the bed) controlling the descent, without coming into contact with the bed itself; the return to the starting position will then be carried out;

- Patient sitting at the edge of the bed, with legs stretched over the Bobath ball (hip flexed at about 90 ° and knees extended): inhalation is combined with flexion of the hips and knees which, by bending, bring the ball close to the patient's body and near the edge of the bed; exhale returning to the starting position;

- With the patient in long-sitting on a carpet, using a Bobath balloon, it will be required to grab the balloon with both hands and upper limbs flexed at 90 ° with elbows extended and bring the balloon first to the right and then to the left, carrying out twisting movements to strengthen the abdominal muscles; this motor sequence will be also performedwith the patient sitting at the edge of the bed with the balloon in front. An anterior flexion of the trunk will then be required associated with a thrust of the ball forward, to the right and then to the left through the use of the upper limbs;

- To strengthen the lateral flexors of the trunk, rhythmic stabilization techniques will be performed, placing the patient on his knees on an oscillating table and keeping the shoulders at 180 ° of flexion with the hands crossed;

- All the exercises proposed for static balance deficits will also be useful for muscle strengthening.

<u>Exercises for proprioception deficits</u>

- Recognition of bags with different contents (sand, pebbles, plastic) by the use of the feet;

- Walking with obstacle courses (such as steps, cushions and bags of different heights and textures) first with open eyes and then with closed eyes, in order to adapt to the different surfaces the feet and then the rest of the body;

- The patient is required to transfer the load from right to left with his feet parallel through the use of a trampoline. The difficulty may be increased by requiring to perform the exercise with eyes closed. In an antero-posterior direction the same load transfer exercise can be required by placing the feet one in front of the other (eg the right front

foot and the left rear foot). These exercises may be proposed on more unstable surfaces such as a multi-axial or mono-axial proprioceptive tablet;

- It can be proposed an exercise for the recovery of the dorsiflexion of the ankle in which the patient have to dorsi-flex the foot and recognize the consistency of several numbered pads that the therapist placed under the heel. This exercise must be performed by the patient sitting at the edge of the bed, with his foot on a board reproduced the flexion-extension movement and with the heel slightly out, keeping his eyes closed, starting from the neutral position of the ankle.

<u>Exercises aimed at improving the gait pattern, coordination and dynamic balance</u>

To improve the gait parameters, stance phase, swing phase

- Step height: paths with obstacles;
- Support base and stepwidth: execution of paths between two rows of pins; walking in tandem along a strip of tape placed on the floor;
- Stride length: walking respecting targets (eg circles);
- Stance phase: in an upright position and with the limb not in load on a stabilization step for maintaining the position; reaching the target with shoulders flexed at 90 °, elbows extended and hands intertwined.
- Swing phase: in an upright position with one limb in charge and the other on a ball, movement on a ball placed under the foot, forward and backward.

<u>Exercises for dynamic balance</u>

- Synergistic work and alternation of agonist and antagonist muscles by asking to walk forward and, at the snap of the fingers, backwards;
- Walking in conjunction with an hand-eye coordination activity: the patient will be asked to walk by bouncing a tennis ball on the floor;
- Walking backwards, thus removing the visual references;
- Walking associated with coordination exercises such as alternately touching the contralateral knee with the upper limb.

<u>Exercises for the improvement of vestibular disorders</u>

- The patient is seated and performs rotational movements with his head in the frontal plane (right and left) and flexion-extension in the sagittal plane (up and down), keeping his gaze fixed on a mobile point (therapist's finger) that moves in the opposite direction with respect to the position of the head;

- The patient will be positioned on a soft texture carpet, bandaged and asked to perform a walk on the spot, keeping his arms flexed at 90 °. The therapist will correct any deviations on the relative planes of space through verbal indications such as "you are turning too far to the right" or "you are going too far", so that the patient can correct himself;

- The patient have to walk on the treadmill, equally blindfolded, gradually varying the speed and asking, at the same time, to rotate the head towards the perceived acoustic stimulus (snap of fingers by the therapist).

<u>Exercises for standing and sitting down ("Sit-to-Stand)</u>

- The patient is sitting on a chair, with his hands resting on a restrained table placed in front of him and with elbows extended. He will be asked to flex the trunk anteriorly, sliding forward with the hands and bringing the nose closer to a target placed on the table. From this position, the extension of the knees is then required in order to lift the pelvis from the chair and then the achievement of the standing position by extending the hips and trunk. The difficulty of the exercise can gradually increase, first by removing the brakes on the table and then completely removing the front support. Once the upright position is reached, the patient is asked to return with his hands resting on the table (in the final phase directly on the thighs), flex the trunk by bringing the nose closer to the target and, again letting the arms slide forward with elbows extended, sit up by guiding the hands in the direction of movement, flexing the knees and controlling the descent.
